# Book Reviews

**Published:** 1895-11

**Authors:** 


### Book Reviews, Etc.

---

A Text-Book of Practical Therapeutics, with Special Reference to the Application of Remedial Measures to Disease and their Employment upon a Rational Basis. By Hobart A. Hare, M. D., B. S. C. Fourth edition. Philadelphia: Lea Bros. Co., 1895

In this day of almost countless new books and revised editions, few are more deservedly popular than "Hare's Therapeutics." The author does not deal in farfetched theories or idly speculate upon the uses of each new and untried drug, but gives to the student or busy practitioner a clear and succinct statement of well-established chemical facts.

Part III. is devoted to "Remedial Measures Other Than Drugs," and gives briefly the uses of hot and cold applications, lavage, suspension, transfusion, etc. Foods for the sick are discussed, giving methods of preparing, together with well-approved diet list.

Part IV. is devoted to "Diseases," and here the student or busy practitioner will find a clear description of the recognized methods of treatment for those diseases met with in every-day practice. Interspersed are prescriptions suited to the conditions described. The entire book is fully abreast of the times, several articles in former editions having been entirely rewritten, and such changes made as are demanded by the light of recent investigations. Inasmuch as the last edition of the "Pharmacopœia" gives only the metric weights and measures, the author has given doses in both the troy and metric systems, placing the two side by side.

In the back of the book are tables giving doses, as well as tables giving relative weights and measures in the metric and apothecary systems.

The subjects treated are arranged alphabetically in the work, thus facilitating reference and obviating the necessity of using an index, though the book is carefully and doubly indexed.

THE MEDICAL NEWS VISITING LIST FOR 1896. Weekly (dated, for 30 patients); Monthly (undated, for 120 patients per month); Perpetual (undated, for 30 patients weekly per year); and Perpetual (undated, for 60 patients weekly per year). The first three styles contain 32 pages of data and 160 pages of blanks. The 60-patient Perpetual consists of 256 pages of blanks. Each style in one wallet-shaped book, with pocket, pencil and rubber. Seal grain leather, $1.25. Philadelphia: Lea Brothers & Co., 1895.

"The Medical News Visiting List for 1896" has been thoroughly revised and brought up to date in every respect. The text portion (32 pages) contains the most useful data for the physician and surgeon, including an alphabetical Table of Diseases, with the most approved Remedies, and a Table of Doses. It also contains sections on Examination of Urine, Artificial Respiration, Incompatibles, Poisons and Antidotes, Diagnostic Table of Eruptive Fevers, and the Ligation of Arteries. The classified blanks (160 pages) are arranged to hold records of all kinds of professional work, with memoranda and accounts. The selection of material in the text portion and the arrangement of the record blanks are the result of eleven years of experience and special study. Equal care has been bestowed upon the mechanical execution of the book, and in quality of paper and in strength and beauty of binding nothing seems to be left wanting. When desired, a Ready Reference Thumb-letter Index is furnished, which is peculiar to this Visiting List, and which will save many-fold its small cost (25 cents) in the economy of time effected during a year. In its several styles "The Medical News Visiting List" adapts itself to any system of keeping professional accounts. In short, every need of the physician seems to have been anticipated in this invaluable pocket companion.

A TREATISE ON NERVOUS AND MENTAL DISEASES. By Landon Carter Gray, M. D., Professor of Diseases of the Mind and Nervous System in the New York Polyclinic. New (2d) edition. In one very handsome octavo volume of 728 pages, with 172 engravings and three colored plates. Cloth, $4.75; leather, $5.75. Philadelphia: Lea Brothers & Co., publishers, 1895.

The second edition of this valuable work has been thoroughly rewritten and a number of chapters added. The reputation of Dr. Gray as a writer and teacher is a sufficient guarantee of the merit of the work. The style is pleasing, almost light, and entirely free from the ponderous verbiage that is so frequently employed in medical text-books, to the clouding of the meaning. The series of illustrations include among others some interesting photographs of the facies of mental disease, and the anatomical diagrams are embellished in black and colors.

The author is one of a long list of Southern men who have risen to prominence and achieved success in the practice of medicine in the North.

TWENTIETH CENTURY PRACTICE: AN INTERNATIONAL ENCYCLOPEDIA OF MODERN MEDICAL SCIENCE. By Leading Authorities of Europe and America. Edited by Thomas L. Stedman, M. D., New York City. In twenty volumes. Volume IV. Diseases of the Vascular System and Thyroid Gland. New York: William Wood and Company, 1895.

The contributors to this volume are Drs. Bertrand Dawson, of London; Murray, of Newcastle-on-Tyne; Sansom, of London, and Whittaker, of Cincinnati. Four hundred and fifty pages are given to the exhaustive article on diseases of the heart and pericardium by Dr. Whittaker.

Diseases of the blood vessels are treated of by Dr. Sansom, and of the lymphatic vessels by Dr. Dawson. The article devoted to diseases of the thyroid gland is particularly in teresting, as it is by Dr. George Murray, to whom is due the credit of introducing the thyroid gland treatment, which has secured such brilliant results in the therapy of myxœdema and cretinism. The article is embellished with photographs illustrating the results of treatment of these curious affections.

The volume is well up to the standard established by the preceding ones.

CLINICAL LECTURES ON DISEASES OF THE NERVOUS SYSTEM, DELIVERED AT THE NATIONAL HOSPITAL FOR THE PARALYZED AND EPILEPTIC, LONDON. By W. R. Gowers, M. D., F. R. S., Philadelphia: P. Blakiston, Son & Co., 1895.

These highly important lectures, thirteen in number, are reprinted from various English medical journals, and in some instances are amplication of the same subjects treated of in the author's well-known work on nervous diseases. The lecture on "mistaken diagnosis" is very instructive. It opens with the wise remark that "it is always a pleasant thing to be right, but it is generally a much more useful thing to be wrong."

A number of deceptive conditions are mentioned which, especially on impartial diagnosis, might be fruitful sources of incorrect diagnosis.

The following is a case in point: "A skilled physician gave orders to his house physician that a surgeon should be called in to tap a chest, one side of which was full of fluid. When the surgeon came, he took notice of the chest before plunging in his trocar, and being a man of thoroughly scientific mind and used to observation, he was struck by the fact that there was no enlargement of the chest. Although he was a surgeon, he percussed carefully, and found that every other sign of plural effusion was present except enlargement of the side. He declined to tap. A little later, the patient died, and the lung was found to be a solid mass of cancer."

The principal subjects treated of, are nervous syphilis, locomator ataxia, syringomyelia, the infantile causes of epilepsy, neuralgia, optic neuritis.

The work offers very entertaining reading, for, being a collection of clinical lectures, it is written in conversational style.

The book is neatly bound in linen and displays the usual typographical excellences of the house from which it is issued.

---

### PUBLISHED ANNUALLY FOR 45 YEARS.—"PHYSICIANS' VISITING LIST FOR 1896."

(Lindsay & Blackiston.)

This well-known visiting list presents several improvements in the new edition for 1896.

More space has been allowed for writing the names and to the "Memoranda Page;" a column has been added for the "Amount" of the weekly visits and a column for the "Ledger Page."

To do this without increasing bulk or price, the reading matter and memoranda pages have been rearranged and simplified.

The lists for 75 patients and 100 patients will also have special memoranda page as above, and hereafter will come in two volumes only, dated January to June, and July to December. While this makes a book better suited to the pocket, the chief advantage is that it does away with the risk of losing the accounts of a whole year should the book be mislaid.

The publishers announce that before making these changes they have personally consulted a number of physicians who have used the book for many years, and have taken into consideration many suggestions made in letters from all parts of the country.

No visiting list has been used to such an extent or for so long a time as this. There is none better suited to the work of the general physician, in keeping easily and systematically his business accounts and memoranda.

